# Effect of Feed Form and Whole Grain Feeding on Gastrointestinal Weight and the Prevalence of *Campylobacter jejuni* in Broilers Orally Infected

**DOI:** 10.1371/journal.pone.0160858

**Published:** 2016-08-08

**Authors:** Marta Isabel Gracia, Jaime Sánchez, Carlos Millán, Óscar Casabuena, Peter Vesseur, Ángel Martín, Francisco Javier García-Peña, Pedro Medel

**Affiliations:** 1 Imasde Agroalimentaria, S.L., Pozuelo de Alarcón, Madrid, Spain; 2 NEPLUVI, Houten, The Netherlands; 3 PROPOLLO, Madrid, Spain; 4 Laboratorio de Sanidad Animal de Algete, MAGRAMA, Algete, Madrid, Spain; Max Rubner-Institut, GERMANY

## Abstract

Two independent trials were carried out to evaluate the effect of feed form, whole wheat (WW) and oat hulls (OH) addition on gastrointestinal (GIT) weight and *Campylobacter jejuni* colonization in orally infected birds. In Trial 1, there were six treatments factorially arranged with two feed forms (mash vs pellets), and three levels of WW from 1-21/22-42d: 0/0, 7.5/15%, 15/30%. Broilers were allocated in cages (3 birds/cage, 12 cages/treatment). In Trial 2, there were three treatments: a mash diet, a mash diet including WW (7.5% from 1–21 and 15% from 22-42d), and a third treatment including also 5%OH. Broilers were allocated in floor pens (1 pen with 30 birds/treatment). At 14d, all broilers in Trial 1 or 3 broilers/pen in Trial 2 were orally challenged with 1.5 x 10^5^ cfu *of C*. *jejuni* ST-45 /. In Trial 1, birds fed pelleted diets consumed 13.5% more feed, gained 31% more weight, and presented 12.9% better feed conversion for the whole trial (P<0.05). Pelleting decreased the relative weight of GIT and gizzard and increased the relative weight of proventriculus (P<0.05). Mash diets decreased pH in the gizzard (P<0.05). Inclusion of WW decreased the relative weight of proventriculus, increased gizzard weight, and reduced pH in the gizzard (P<0.05). At 21d of age, mash tended to reduce *C*. *jejuni* compared to pellets (7.85 vs 8.27 log_10_cfu/g; P = 0.091) and WW inclusion at 7.5/15% reduced *C*. *jejuni* colonization when compared to lower and higher inclusion (P<0.05). In Trial 2, birds fed T3 (WW+OH) showed 1.38 log_10_cfu/g less than birds fed Control diet (P<0.05). In conclusion, despite of the clear morphological changes in the GIT derived of FF and WW inclusion, no clear reductions in *C*. *jejuni* populations in the ceca were observed. However, WW and OH inclusion to mash diets significantly reduced cecal *C*. *jejuni* colonization at 42 days.

## Introduction

*Campylobacter* is the leading cause of bacterial gastroenteritis in humans worldwide. In the EU, it is the most commonly reported gastrointestinal bacterial pathogen since 2005, with 236,851 human cases reported in 2014 [1].

*Campylobacter* spreads rapidly within broiler flocks through horizontal transmission so that the prevalence within the flock may increase from <5% to >95% in a week [2–4]. The principal site of colonization is the lower gastrointestinal tract (**GIT**), especially in the ceca [5–8]. During slaughter, positive broiler flocks can cause carcass contamination [9], that may serve as a source for cross-contamination to other foodstuffs and surfaces during meal preparation in the consumer's kitchen [10,11]. Therefore, implementation of *Campylobacter* control measures at the primary production level is needed to reach a reduction of human campylobacteriosis.

Some intervention efforts targeting the lower GIT have been evaluated in order to reduce the colonization of *C*. *jejuni* in poultry [12]. However, few efforts have been done trying to modify also the upper GIT in order to combat *C*. *jejuni* colonization in lower GIT. Inclusions of oat hulls (**OH**) or whole wheat (**WW**) in feed have been shown to modify the upper GIT of broilers, with increased gizzard weights and lower pH levels [13]. Also, it should be considered that better gizzard activity might promote nutrient digestibility, leaving less nutrients available for bacterial proliferation in the intestine [14], with beneficial microbiological consequences in the lower GIT. For example, dietary inclusion of WW has been shown to decrease intestinal *Salmonella* colonization [15,16] and *Clostridium perfringens* counts [15,17] in broilers. Gabriel et al. [14] also reported that birds fed WW had higher counts of beneficial microflora and lower counts of coliform bacteria. Moen et al. [18] have also shown that a stimulated gizzard through oat/barley hulls inclusion delays the horizontal spread of *C*. *jejuni* in broiler flocks. Similarly, Skånseng et al. [19] found a delay in the spread and a reduction in the amount of *C*. *jejuni* in cecum of chickens fed WW compared to chickens given control feed. These results indicate that a functional gizzard may act as a barrier organ preventing potential pathogenic bacteria from entering the distal digestive tract. In addition, a well-developed GIT enhances motility, favors gastroduodenal refluxes, and stimulates the secretion of enzymes and functionality which might affect microbial profile and health status of the birds.

Therefore, the objective of this work was to study the effects of different feed form, WW and dietary insoluble fiber inclusion on GIT development and consequently on the reduction of cecal colonization of *Campylobacter* in broilers.

## Materials and Methods

### Experimental Design

Two independent trials were carried out to test feed form, WW and OH inclusion. A total of 306 day-of-hatch Ross 308 broilers chicks (50% male and 50% female) were used (n = 36 chicks per treatment in Trial 1 and n = 30 chicks per treatment in Trial 2). Trial 1 consisted in six treatments arranged factorially with two feed forms (mash vs pellets) and three levels of WW inclusion (1-21d/22-42d: 0/0%, 7.5/15% and 15/30%). In trial 2, there were three treatments, a mash control diet, a mash diet including WW at 7.5% from 1-21d and 15% from 22-42d, and a third treatment including also 5% OH (Table 1). Trials were carried out in a facility with Animal Biosafety Level 2. In Trial 1, the birds were kept in wire-floored cages in groups of three birds with an area of 0.21 m^2^ (0.50 m x 0.42 m). In trial 2 the birds were kept in three floor pens, in groups of 30 birds, with an area of 1.83 m^2^ (1.58 m x 1.16 m) and fresh wood shavings as bedding material. The building was supplied with artificial, programmable lights (18 hours light and 6 hour dark during each 24-hour period), automated electric heating and forced ventilation. The experimental diets consisted of standard non-medicated, non-coccidiostats, cereal (wheat/corn)-soy based diets (Tables 2 and 3). The starter diet was offered to birds from 1 day-old until 21 days of age and finisher diet from day 22 to 42 days. Feed and water were available *ad libitum*. Crude protein, fat, crude fiber, starch, calcium and total phosphorus were analyzed according to [20] procedures. This study was carried out in strict accordance with the recommendations in the Guide for the Care and Use of Agricultural Animals in Research and Teaching [21]. Animals were monitored twice daily in their pens. No animals became ill or died during the trial. Husbandry, euthanasia methods, experimental procedures and biosafety precautions were approved by the Research Ethical Committee of the University of Murcia. Broiler weights and feed intake were determined per cage in Trial 1 at 21 and 42 days of age, and daily gain, feed consumption and feed:gain ratio calculated at 1–21, 22–42 and 1–42 d. Also in Trial 1, the weight of gastrointestinal tract (GIT, from the proventriculus to the cloaca), proventriculus (whole and empty), gizzard (whole and empty), and ceca were taken using a scale (Gram Precision, Barcelona, Spain) at 42 days from 12 broilers per treatment. Also the pH at proventriculus, gizzard and caeca were measured (pH-meter Crison, Barcelona, Spain).

**Table 1:** Experimental treatments in Trials 1 and 2.

Trial	Treatment	Feed form	Whole wheat (WW) inclusion, %		Oat hulls (OH) inclusion, %
			1–21 days	22–42 days	1–42 days
Trial 1	T1	Mash	0	0	0
	T2	Mash	7.5	15	0
	T3	Mash	15	30	0
	T4	Pellet	0	0	0
	T5	Pellet	7.5	15	0
	T6	Pellet	15	30	0
Trial 2	T1	Mash	0	0	0
	T2	Mash	7.5	15	0
	T3	Mash	7.5	15	5

**Table 2:** Formulation, calculated and analyzed composition of experimental diets in Trial 1, as fed basis.

Ingredients (g/kg)	1–21 d			22–42 d		
	Basal	92.5% Basal + 7.5% WW	85% Basal + 15% WW	Basal	85% Basal + 15% WW	70% Basal + 30% WW
Corn	72.6	67.2	61.7	106.9	90.9	74.8
Wheat	500.0	462.5	425.0	500.0	425.0	350.0
Soybean meal (47%, CP)	252.9	234.0	215.0	186.2	158.2	130.3
Full fat soybean	109.1	100.9	92.7	136.3	115.9	95.4
Palm oil	20.0	18.5	17.0	30.0	25.5	21.0
Calcium carbonate	10.0	9.3	8.5	9.5	8.1	6.6
Dicalcium phosphate	20.0	18.5	17.0	17.1	14.6	12.0
Salt	2.0	1.8	1.7	2.1	1.8	1.5
Sodium bicarbonate	2.4	2.2	2.0	2.3	1.9	1.6
DL-Methionine	3.4	3.1	2.9	2.7	2.3	1.9
L-Lysine HCL	2.5	2.3	2.1	2.1	1.8	1.5
L-Threonine	1.1	1.0	0.9	0.8	0.6	0.5
Premix^a^	4.0	3.7	3.4	4.0	3.4	2.8
Whole wheat (WW)	0	75.0	150.0	0	150.0	300.0
**Calculated nutrients (g/kg)**^**b**^						
AME_n_ (kcal/kg)	2,950	2,961	2,973	3,075	3,079	3,083
Crude protein	220.0	211.2	202.3	200.0	185.3	170.6
Ether extract	56.2	53.2	50.2	71.3	63.0	54.7
Crude fibre	31.7	31.3	30.8	31.4	30.6	29.8
Starch	346.0	365.2	384.4	366.3	401.6	437.0
Calcium	10.0	9.3	8.6	9.0	7.7	6.5
Available phosphorus	4.5	4.3	4.1	4.0	3.6	3.3
Digestible lysine	11.8	11.1	10.4	10.4	9.2	8.0
Digestible methionine + cystine	9.2	8.8	8.3	8.1	7.4	6.7
Digestible threonine	7.7	7.3	6.9	6.7	6.0	5.4
Digestible tryptophan	2.3	2.2	2.1	2.1	1.9	1.8
**Feed analysis (g/kg)**						
Crude protein	230	218	210	212	193	175
Ether extract	52	49	47	66	60	55
Crude fibre	33	35	35	36	38	36
Starch	363	329	353	372	370	418
Calcium	10.6	11.1	10.1	11.3	10.0	8.7
Total phosphorus	6.3	5.9	6.0	6.4	5.5	5.2

^a^Premix provided per kg of basal diet: Vitamin A (E 672): 10,000 IU; Vitamin D3 (E 671): 2,000 IU; Vitamin E (α-tocopherol): 30.0 mg; Vitamin K3: 2.0 mg; Vitamin B1: 2.0 mg; Vitamin B2: 5.0 mg; Vitamin B6: 3.0 mg; Vitamin B12: 12.0 μg; Nicotinic acid: 40.0 mg; Calcium pantothenate: 10.0 mg; Folic acid: 1.0 mg; Biotin: 0.1 mg; Choline chloride: 400 mg; Cu (CuSO_4_·5H_2_O): 8.0 mg; Fe (FeCO_3_): 60.0 mg; I (IK): 2.0 mg; Mn (MnO): 70.0 mg; Se (Na_2_SeO_3_): 0.15 mg; Zn (ZnO): 80.0 mg.

^b^According to FEDNA [22].

**Table 3:** Formulation, calculated and analyzed composition of experimental diets in Trial 2, as fed basis.

Ingredients (g/kg)	1–21 d			22–42 d		
	Basal	92.5% Basal + 7.5% WW	87.5% Basal + 7.5% WW + 5% OH	Basal	85% Basal + 15% WW	80% Basal + 15% WW + 5% OH
Corn	72.6	67.2	63.6	106.9	90.9	85.5
Wheat	500.0	462.5	437.5	500.0	425.0	400.0
Soybean meal (47%, CP)	252.9	234.0	221.3	186.2	158.2	148.9
Full fat soybean	109.1	100.9	95.5	136.3	115.9	109.1
Palm oil	20.0	18.5	17.5	30.0	25.5	24.0
Calcium carbonate	10.0	9.3	8.8	9.5	8.1	7.6
Dicalcium phosphate	20.0	18.5	17.5	17.1	14.6	13.7
Salt	2.0	1.8	1.7	2.1	1.8	1.7
Sodium bicarbonate	2.4	2.2	2.1	2.3	1.9	1.8
DL-Methionine	3.4	3.1	3.0	2.7	2.3	2.2
L-Lysine HCL	2.5	2.3	2.2	2.1	1.8	1.7
L-Threonine	1.1	1.0	1.0	0.8	0.6	0.6
Premix^a^	4.0	3.7	3.5	4.0	3.4	3.2
Whole wheat (WW)	0.0	75.0	75.0	0.0	150.0	150.0
Oat hulls (OH)	0.0	0.0	50.0	0.0	0.0	50.0
**Calculated nutrients (g/kg)**^**b**^						
AME_n_ (kcal/kg)	2,950	2,961	2,834	3,075	3,079	2,945
Crude protein	220.0	211.2	202.1	200.0	185.3	177.2
Ether extract	56.2	53.2	51.1	71.3	63.0	60.2
Crude fibre	31.7	31.3	44.7	31.4	30.6	44.1
Starch	346.0	365.2	352.2	366.3	401.6	387.7
Calcium	10.0	9.3	8.8	9.0	7.7	7.3
Available phosphorus	4.5	4.3	4.1	4.0	3.6	3.4
Digestible lysine	11.8	11.1	10.5	10.4	9.2	8.7
Digestible methionine + cystine	9.2	8.8	8.3	8.1	7.4	7.0
Digestible threonine	7.7	7.3	6.9	6.7	6.0	5.7
Digestible tryptophan	2.3	2.2	2.1	2.1	1.9	1.8
**Feed analysis (g/kg)**						
Crude protein	230	220	213	212	187	187
Ether extract	52	51	48	66	59	59
Crude fibre	33	36	42	36	33	46
Starch	363	350	348	372	386	375
Calcium	10.6	11.6	12.1	11.3	10.7	9.4
Total phosphorus	6.3	5.8	6.0	6.4	5.8	5.3

^a^Premix provided per kg of basal diet: Vitamin A (E 672): 10,000 IU; Vitamin D3 (E 671): 2,000 IU; Vitamin E (α-tocopherol): 30.0 mg; Vitamin K3: 2.0 mg; Vitamin B1: 2.0 mg; Vitamin B2: 5.0 mg; Vitamin B6: 3.0 mg; Vitamin B12: 12.0 μg; Nicotinic acid: 40.0 mg; Calcium pantothenate: 10.0 mg; Folic acid: 1.0 mg; Biotin: 0.1 mg; Choline chloride: 400 mg; Cu (CuSO_4_·5H_2_O): 8.0 mg; Fe (FeCO_3_): 60.0 mg; I (IK): 2.0 mg; Mn (MnO): 70.0 mg; Se (Na_2_SeO_3_): 0.15 mg; Zn (ZnO): 80.0 mg.

^b^According to FEDNA [22].

### Broiler Inoculation

The strain *C*. *jejuni* ST-45 was isolated from Spanish broiler flocks in a prevalence study carried out in 2007–2008, characterized (hippurate hydrolysis test positive, PCR [23,24] and MLST typed) and is stored at -70°C in peptone broth containing 20% (vol/vol) glycerol in Laboratorio Central de Veterinaria (Algete, Spain). Five days before each inoculation, the strain was recovered from frozen stock after plating on mCCDA (selective modified Charcoal Cefoperazone Deoxycholate Agar) at 42°C for 48 h under a microaerophilic atmosphere (85% N_2_, 10% CO_2_ and 5% O_2_). Cells were harvested and diluted to an appropriate density of 1.5 x 10^8^ cfu/mL using a spectrophotometer and standard curve. Finally, 0.1 mL is diluted into 10 mL in tryptone salt broth, with a final concentration of 1.5 x 10^6^ cfu/mL.

In trial 1, all the animals were orally challenged by crop instillation at 14 days of age with 1.5 x 10^5^ cfu of *Campylobacter jejuni* ST-45 (0.1 mL). Oral administration of 0.1 ml/bird of the bacterial suspension was performed by instillation into the crop using a syringe with an attached flexible tube. In trial 2, three broilers per pen selected at random were orally challenged following the same procedure.

### Sampling and Microbiological Analysis

Prior to oral infection with *C*. *jejuni*, two broiler chicks per treatment were randomly selected and euthanized in order to check the absence of *C*. *jejuni* infection in the ceca. Analyses were carried out according to the ISO 10272 standard, direct plating of 1g of cecal content. The confirmation of the concentration of *Campylobacter* in the administered inoculum was also determined after the oral infection in each trial by serial dilution, and plating 0.1 mL of each dilution on mCCDA. On days 21, 35 and 42 of age (corresponded to 7, 21 and 28 days post challenge, respectively) in Trial 1 and on days 21 and 42 of age (corresponded to 7 and 28 days post challenge, respectively) in Trial 2, 12 (Trial 1) or 10 birds (Trial 2) from each treatment group were euthanized and immediately the ceca aseptically removed. Cecal contents from each bird were homogenized and 1 g diluted 1/10 (wt/vol) in tryptone salt broth within 1 hour after euthanasia. After homogenization, enumeration was carried out in duplicate by serial dilution in tryptone salt broth in order to assess *C*. *jejuni* count on mCCDA plates after 44 ± 4 h of incubation at 42 ± 1°C in a microaerophilic atmosphere (85% N_2_, 10% CO_2_ and 5% O_2_). The detection limit for enumeration of the *Campylobacter* was 1 x 10^2^ cfu/g of cecal content.

### Statistical Analysis

Data were analyzed by IBM SPSS 19.0 Statistics for Windows Version 19.0. (IBM Corp., Armonk, NY). The individual logarithms of the 12 or 10 birds per group and sampling age of each experiment were used as experimental unit for statistical analysis. For performance data, the cage was used as the experimental unit. The Shapiro-Wilk test was used to test the normal distribution of the data. As the distribution of data was normal, data were analyzed as a completely randomized design through analysis of variance (ANOVA) followed by the Tukey’s test to find the significance between main effects (Trial 1) or treatments (Trial 2). In Trial 1, the model included feed form and WW inclusion as main effects and their interaction. In Trial 2, the model included the experimental treatment as main effect. Statistical significance was declared at P ≤ 0.05, with 0.05 < P ≤ 0.10 considered as a near-significant trend.

## Results

### Performance

Broilers fed pelleted diets gained more weight than broilers fed mash diets (33.0 vs 44.8 g/d, 67.6 vs 87.6 g/d, and 49.4 vs 64.8 g/d for mash vs pelleted diets from 1–21 d, 22–42 d, and 1–42 d, respectively; P < 0.05) (Table 4). The better growth observed in chickens fed pelleted diets was due to a higher feed consumption during the whole fattening period (95.2 vs 108.1 g/d from 1-42d for mash vs pelleted diets; P < 0.05). Feed:gain ratio was also improved by pelleting (1.93 vs 1.68 g/g from 1–42 d for mash vs pelleted diets, P < 0.05). WW inclusion impaired feed:gain ratio at 1–21 d, but no significant effects in performance (daily gain, feed consumption and feed:gain ratio) were observed for the whole trial (1–42 d). With regard to the use of WW, a selective uptake of the raw material might have occurred, resulting in an important issue relating to the interpretation of the results. Selectivity on the consumption was not specifically measured, but no refusal of whole wheat was observed, and feeds with WW were consumed in the same proportion than feed, as no differences between original feed with WW and feeder refusals were observed at 21, 35 and 42 days.

**Table 4:** Mean daily gain, feed consumption and feed:gain ratio at 1–21, 22–42 and 1–42 d of age in in Trial 1.

Treatment		Whole wheat inclusion (1-21/22-42 d)			Mean
		0%/0%	7.5%/15%	15%/30%	
Mean daily gain 1-21g (g)					
	Mash feeds	33.3	32.6	33.2	33.0^b^
	Pelleted feeds	45.3	43.9	45.1	44.8^a^
	Mean	39.3	38.3	39.1	1.23^1^
Feed consumption 1-21d (g/d)					
	Mash feeds	51.3	55.8	55.3	54.2^b^
	Pelleted feeds	66.6	70.4	73.2	70.1^a^
	Mean	59.0	63.1	64.3	2.50^1^
Feed:gain ratio 1-21d					
	Mash feeds	1.55	1.72	1.69	1.65^x^
	Pelleted feeds	1.47	1.60	1.63	1.57^y^
	Mean	1.51^b^	1.66^a^	1.66^a^	0.05^1^
Mean daily gain 22-42g (g)					
	Mash feeds	67.9	63.8	71.1	67.6^b^
	Pelleted feeds	88.1	88.1	86.5	87.6^a^
	Mean	78.0	76.0	78.8	3.38^1^
Feed consumption 22-42d (g/d)					
	Mash feeds	132.0	138.8	138.4	136.4^b^
	Pelleted feeds	152.0	143.7	144.7	146.8^a^
	Mean	142.0	141.2	141.5	5.58^1^
Feed:gain ratio 22-42d					
	Mash feeds	1.95	2.17	1.98	2.03^a^
	Pelleted feeds	1.73	1.66	1.70	1.70^b^
	Mean	1.84	1.92	1.84	0.06^1^
Mean daily gain 1-42g (g)					
	Mash feeds	49.6	47.1	51.6	49.4^b^
	Pelleted feeds	65.6	64.6	64.2	64.8^a^
	Mean	57.6	55.8	57.9	1.89^1^
Feed consumption 1-42d (g/d)					
	Mash feeds	91.6	96.7	97.3	95.2^b^
	Pelleted feeds	109.4	106.0	109.0	108.1^a^
	Mean	100.5	101.4	103.1	3.20^1^
Feed:gain ratio 1-42d					
	Mash feeds	1.85	2.06	1.90	1.93^a^
	Pelleted feeds	1.67	1.65	1.71	1.68^b^
	Mean	1.76	1.85	1.81	0.04^1^

^a,b/x,y^Means within a main effect with different superscripts are significantly different (a, b: P<0.05; x, y: 0.05<P<0.10).

^1^Pooled standard error of the mean (SEM, n = 12).

### GIT weight

In general, feed form affected GIT weight more than WW inclusion (Table 5). Pelleting decreased the GIT weight expressed as percentage of BW (8.47 vs 7.78% for mash vs pelleted diets; P < 0.05). Pelleting the diet increased the weight of the proventriculus in relation to the GIT (4.54 vs 5.76% GIT for mash vs pelleted diets; P < 0.05) but decreased the relative weight of the gizzard expressed as percentage of BW and GIT (2.40 vs 1.96% BW and 28.52 vs 24.95% GIT for mash vs pelleted diets; P < 0.05). Also, the weight of empty gizzard in relation to BW was reduced by pelleting (1.69 vs 1.37% BW for mash vs pelleted diets; P<0.05). No significant effects of pelleting were observed in ceca weight.

**Table 5:** Gastrointestinal tract weight at 42 d of age in Trial 1.

Treatment		Whole wheat inclusion (1-21/22-42 d)			Mean
		0%/0%	7.5%/15%	15%/30%	
GIT weight (% BW)					
	Mash feeds	8.26	8.59	8.55	8.47^a^
	Pelleted feeds	7.59	7.69	8.06	7.78^b^
	Mean	7.92	8.14	8.31	0.30^1^
Proventriculus (% BW)					
	Mash feeds	0.39	0.37	0.38	0.38
	Pelleted feeds	0.68	0.35	0.35	0.46
	Mean	0.54^a^	0.36^b^	0.37^b^	0.16^1^
Proventriculus (% GIT)					
	Mash feeds	4.81	4.37	4.45	4.54^b^
	Pelleted feeds	8.22	4.68	4.37	5.76^a^
	Mean	6.51^a^	4.52^b^	4.41^b^	1.68^1^
Empty proventriculus (% BW)					
	Mash feeds	0.32	0.33	0.35	0.33
	Pelleted feeds	0.42	0.30	0.33	0.35
	Mean	0.37	0.31	0.34	0.03^1^
Digesta proventriculus (% Prov.)					
	Mash feeds	18.72	11.23	8.57	14.65
	Pelleted feeds	29.31	13.14	7.54	16.25
	Mean	24.01^a^	12.18^b^	8.05^b^	3.87^1^
Gizzard (% BW)					
	Mash feeds	2.16	2.46	2.58	2.40^a^
	Pelleted feeds	1.39	2.21	2.27	1.96^b^
	Mean	1.78^b^	2.33^a^	2.42^a^	0.11^1^
Gizzard (% GIT)					
	Mash feeds	26.23	28.88	30.46	28.52^a^
	Pelleted feeds	17.72	28.93	28.21	24.95^b^
	Mean	21.98^b^	28.90^a^	29.34^a^	1.06^1^
Empty gizzard (% BW)					
	Mash feeds	1.42	1.74	1.90	1.69^a^
	Pelleted feeds	1.06	1.51	1.55	1.37^b^
	Mean	1.24^b^	1.63^a^	1.72^a^	0.06^1^
Digesta gizzard (% Gizzard)					
	Mash feeds	33.75	28.58	26.45	29.59
	Pelleted feeds	17.36	30.54	31.81	26.57
	Mean	25.55	29.56	29.13	2.63^1^
Ceca (% BW)					
	Mash feeds	0.71	0.92	0.66	0.76
	Pelleted feeds	0.65	0.69	0.77	0.70
	Mean	0.68	0.80	0.71	0.07^1^
Ceca (% GIT)					
	Mash feeds	8.54	10.67	7.74	8.98
	Pelleted feeds	8.70	8.89	9.36	8.99
	Mean	8.62	9.78	8.55	0.81^1^

^a,b^Means within a main effect with different superscripts are significantly different (P<0.05).

^1^Pooled standard error of the mean (SEM, n = 12).

WW inclusion decreased the weight of the proventriculus in relation to BW (0.54, 0.36 and 0.37% BW for 0/0, 7.5/15 and 15/30% WW, respectively; P < 0.05) and to the GIT (6.51, 4.52 and 4.41% GIT for 0/0, 7.5/15 and 15/30% WW, respectively; P < 0.05). On the contrary, WW inclusion clearly increased the weight of the gizzard in relation to BW (1.78, 2.33 and 2.42% BW for 0/0, 7.5/15 and 15/30% WW, respectively; P < 0.05) and to the GIT (21.98, 28.90, and 29.34% GIT for 0/0, 7.5/15 and 15/30% WW, respectively; P < 0.05). A similar increase with WW inclusion was observed in the weight of the empty gizzard related to BW. Also, WW inclusion decreased the amount of fresh digesta in the proventriculus (24.01, 12.18 and 8.05% proventriculus for 0/0, 7.5/15 and 15/30% WW, respectively; P < 0.05) but did not affect the content of gizzard. No significant effects of WW inclusion were observed in ceca weight.

**Fig 1:**
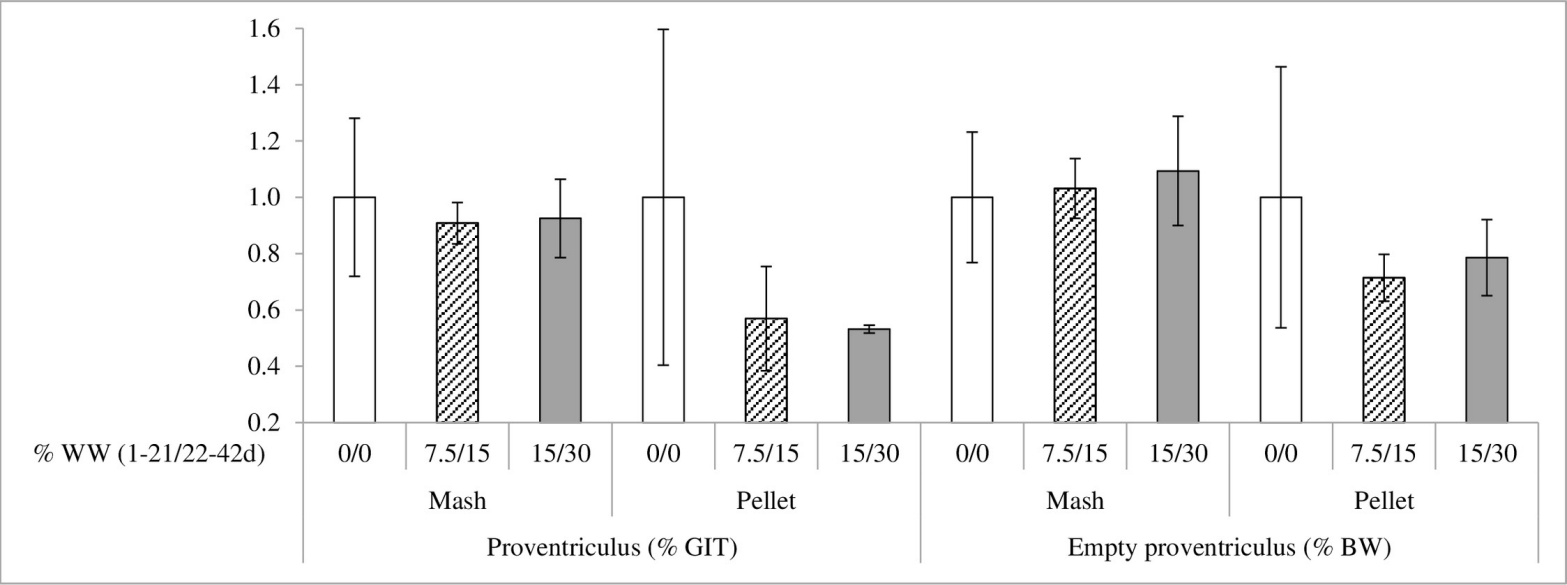
Interaction between feed form and whole wheat (WW) inclusion in proventriculus weight (% GIT) and empty proventriculus weight (% BW) (P<0.05). Relative weight of the proventriculus (% GIT) and empty proventriculus weight (% BW) of 42 days broilers fed either mash or pelleted feeds and different levels of whole wheat (WW) at 0-21/21-42 days: 0/0% designated with white bars, 7.5/15% designated with shaded bars and 15/30% designated with dark bars. Results are expressed in relation the 0/0% inclusion level.

There was an interaction between feed form and WW inclusion for proventriculus weight in relation to the GIT and for the empty proventriculus weight in relation to BW (P < 0.05). WW inclusion clearly reduced the proventriculus size in pelleted diets, but not in mash diets (Fig 1). In fact, birds fed with pelleted diets without WW, showed proventricultis, which was counteracted by WW inclusion.

**Fig 2:**
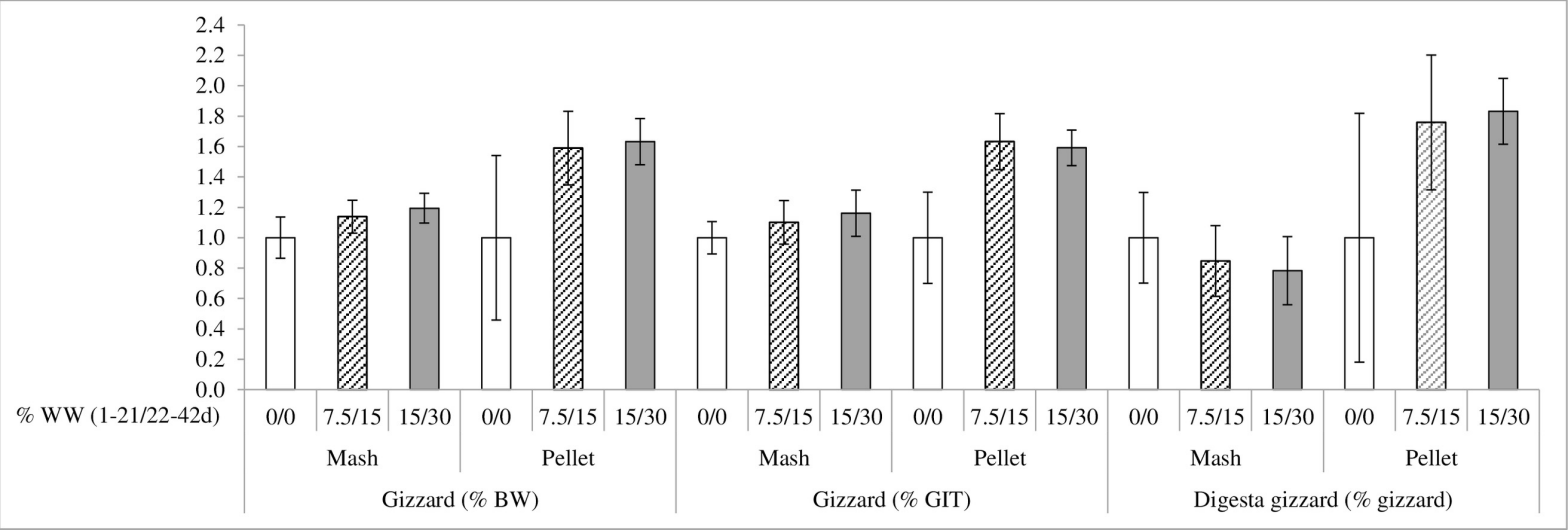
Interaction between feed form and whole wheat (WW) inclusion in gizzard weight (% BW and % GIT) and digesta content of gizzard (% gizzard) (P<0.05). Relative weight of the gizzard (% BW and % GIT) and digesta content of gizzard (% gizzard weight) of 42 days broilers fed either mash or pelleted feeds and different levels of whole wheat (WW) at 0-21/21-42 days: 0/0% designated with white bars, 7.5/15% designated with shaded bars and 15/30% designated with dark bars. Results are expressed in relation the 0/0% inclusion level.

There was also an interaction between feed form and WW inclusion for gizzard weight in relation to BW and GIT and for the content of the gizzard in relation to the gizzard weight (P < 0.05). WW inclusion increased the size of the gizzard and the amount of digesta in the gizzard in pelleted diets but not in mash diets (Fig 2).

### pH of intestinal contents

**Fig 3:**
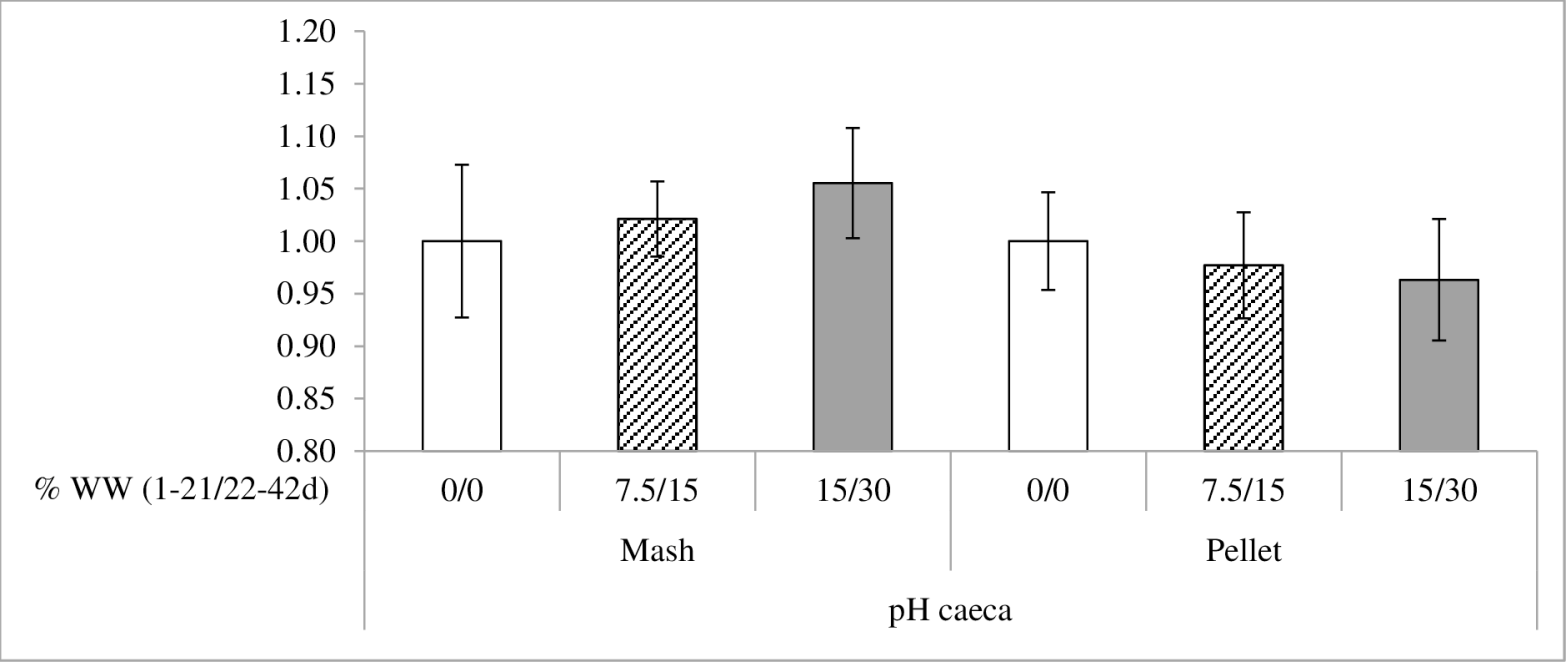
Interaction between feed form and whole wheat (WW) inclusion in pH at ceca (P<0.05). pH at ceca of 42 days broilers fed either mash or pelleted feeds and different levels of whole wheat (WW) at 0-21/21-42 days: 0/0% designated with white bars, 7.5/15% designated with shaded bars and 15/30% designated with dark bars. Results are expressed in relation the 0/0% inclusion level.

Differences in pH were only observed in the gizzard (Table 6). Mash diets decreased pH values in the gizzard when compared to pelleted diets (3.04 vs 3.52 for mash vs pelleted diets; P < 0.05). Also, WW inclusion decreased pH in the gizzard (3.48, 3.29 and 3.07 for 0/0, 7.5/15 and 15/30% WW, respectively; P < 0.05). There was an interaction between feed form and WW inclusion for pH in the ceca (P < 0.05). WW inclusion increased pH in mash diets but it was reduced in pelleted diets (Fig 3).

**Table 6:** pH at proventriculus, gizzard and ceca at 42 d of age in in Trial 1.

Treatment		Whole wheat inclusion (1-21/22-42 d)			Mean
		0%/0%	7.5%/15%	15%/30%	
pH proventriculus					
	Mash feeds	3.32	3.58	3.69	3.53
	Pelleted feeds	4.00	3.83	3.53	3.79
	Mean	3.66	3.70	3.61	0.94^1^
pH gizzard					
	Mash feeds	3.13	3.02	2.96	3.04^b^
	Pelleted feeds	3.83	3.57	3.17	3.52^a^
	Mean	3.48^1^	3.29^a^^b^	3.07^b^	0.13^1^
pH ceca					
	Mash feeds	6.15	6.28	6.49	6.31
	Pelleted feeds	6.52	6.37	6.28	6.39
	Mean	6.33	6.33	6.38	0.10^1^

^a,b^Means within a main effect with different superscripts are significantly different (P<0.05).

^1^Pooled standard error of the mean (SEM, n = 12).

### *C*. *jejuni* colonization

The bacteriological analysis of cecal samples collected from the broilers before *C*. *jejuni* challenge demonstrated that broiler chicks were free of *Campylobacter* spp. Results are presented in Tables 7 and 8 for Trials 1 and 2, respectively. In Trial 1, mash diets tended to reduce *C*. *jejuni* colonization when compared to pelleted diets at 7 d post challenge (7.65 vs 8.27 log_10_cfu/g; P = 0.091). Also at this age, WW inclusion at 7.5/15% reduced *C*. *jejuni* colonization when compared to lower and higher inclusion (7.33 vs 8.32 and 8.23 log_10_cfu/g, for 7.5/15 vs 0/0 and 15/30%, respectively; P < 0.05). No significant differences in cecal *Campylobacter* counts of broilers at 21 or 28 d post challenge were observed with feed form of WW inclusion. In Trial 2, no significant differences between treatments were observed in cecal *Campylobacter* counts at 7 d post challenge. However, the supplementation of 5% OH together with WW inclusion at 7.5/15% reduced cecal *C*. *jejuni* colonization by 1.38 log_10_cfu/g in relation to Control diet and 0.90 log_10_cfu/g in relation to WW inclusion at 28 d post challenge (8.10, 9.48 and 9.00 log_10_cfu/g for WW+OH, Control and WW, respectively; P < 0.05). The colonization level in this trial was very high, however and even with the significant reduction obtained with WW and OH, the final contamination level was above acceptable limits.

**Table 7:** Effect of feed form and whole wheat (WW) inclusion on the cecal colonization of *C*. *jejuni* (log_10_ cfu/g cecal content) in broilers chickens in Trial 1^1^.

Treatment		WW inclusion (1-21/22-42 d)			Mean
		0%/0%	7.5%/15%	15%/30%	
7 d post-challenge					
	Mash feeds	8.17	6.88	7.89	7.65^y^
	Pelleted feeds	8.47	7.78	8.57	8.27^x^
	Mean	8.32^a^	7.33^b^	8.23^a^	0.45^2^
21 d post-challenge					
	Mash feeds	7.25	6.88	6.55	6.89
	Pelleted feeds	7.04	7.11	6.85	7.00
	Mean	7.15	6.99	6.70	0.48^2^
28 d post-challenge					
	Mash feeds	6.64	7.00	6.20	6.61
	Pelleted feeds	6.03	6.30	6.14	6.16
	Mean	6.33	6.65	6.17	0.72^2^

^a,b/x,y^Means within a main effect with different superscripts are significantly different (a, b: P<0.05; x, y: 0.05<P<0.10).

^1^Data presented as means of logarithms of 12 cecal samples per group (log_10_ cfu/g).

^2^Pooled standard error of the mean (SEM, n = 12).

**Table 8:** Effect of whole wheat (WW) and oat hulls (OH) addition to a basal diet on the cecal colonization of *C*. *jejuni* (log_10_ cfu/g cecal content) in broilers chickens in Trial 2^1^.

Treatment		7 d post challenge	28 d post challenge
1	Basal diet	9.36	9.48^a^
2	Basal diet + 7.5/15.0% WW	9.19	9.00^a^
3	Basal diet + 7.5/15.0% WW + 5% OH	9.00	8.10^b^
SEM^2^		0.222	0.240
Probability		0.527	**0.001**

^a, b^Means within the same column with different superscripts are significantly different (P<0.05).

^1^Data presented as means of logarithms of 10 cecal samples per group (log_10_ cfu/g).

^2^Standard error of the mean.

## Discussion

The aim of this research was to search for feeding strategies able to reduce cecal colonization of *Campylobacter* in broilers at slaughter age *in vivo*. For that purpose, different WW levels and OH were included into mash or pelleted feeds. The better performance of broilers fed pelleted diets relative to those fed mash diets is well known in poultry production, with improved weight gain, feed intake, and feed efficiency in broilers regardless grain source [25,26]. In fact, pelleting usually increases feed intake of broiler chickens by 10 to 20% [27,28]. Our performance results are in agreement with previous research as pelleting improved growth by 31.2%, feed intake by 13.6% and feed efficiency by 12.9% during the whole experiment. Most studies involving WW inclusion have evaluated post-pelleting inclusion of WW and all show no adverse effects on weight gain [28–31]. On the other hand, other studies reported negative effects on feed per gain ratio [32–34]. Similar results have been obtained in our work, with no adverse effects of WW inclusion in weight gain and feed per gain in the whole experiment, even with the higher level of dilution, but showing negative effects on feed per gain at 1–21 days.

Broilers fed pelleted diets had increased proventriculus weight and decreased gizzard weight as compared to those fed mash diets. Under current commercial feeding regimes, birds fed finely ground, pelleted diets show dilation of the proventriculus and a relatively underdeveloped gizzard [35–40], which functions as a transit rather than a grinding organ [41].

The opposite occurred with WW inclusion, which decreased proventriculus weight and increased gizzard weight as compared to those without WW. Singh et al. [42] reported that most studies evaluating post-pelleting inclusion of WW found increased gizzard weights. The development of the gizzard with WW feeding is a response to increased frequency of contraction to reduce whole grains to fine particles [43,44]. Engberg et al. [17] also reported increased gizzard weight with WW addition. Indeed, Gabriel et al. [40] reported smaller proventriculus and larger gizzards with increasing replacement of ground wheat by WW in broilers from 8 to 44 days.

In the present experiment, WW inclusion clearly reduced the proventriculus size in pelleted diets, but not in mash diets, because birds fed with pelleted diets without WW showed a clear proventriculitis, that was counteracted by WW inclusion. The same effect was described by Taylor and Jones [45,46] where hypertrophy of the proventriculus in broilers fed pelleted diets was completely eliminated by whole grain feeding.

WW inclusion increased the size of the gizzard in pelleted diets but not in mash diets. The higher feed intake produced with pelleting may therefore have particularly detrimental effects when no structural components exist in the diet, resulting in a small and under-developed gizzard; effect that was reverted with WW inclusion.

As summarized by Svihus [13], most of the recent average values recorded for the pH of the gizzard of broiler chickens are reported to be between 3 and 4 for normal pelleted diets, being the values obtained in our research on the reported average. The pH of gizzard content was higher in pelleted than in mash diets. Svihus [47] hypothesized that as feed usually has a pH close to neutral, higher feed intake may result in an elevated gizzard pH, being probably the main reason why gizzard pH is reported to be higher with pelleted diets compared with mash diets [27,48,49], although less particle size due to the grinding effect of pelleting might also have contributed to this effect [27,28].

The pH of gizzard content decreased with WW inclusion. Svihus [13] reported that when structural components, such as whole or coarsely ground cereals, or fiber materials, such as hulls or wood shavings, are added, the pH of the gizzard content decreases by a magnitude of 0.2 to 1.2 units. The decrease obtained in the present work varies from 0.11 in mash diets to 0.66 in pelleted feeds, which agrees with previous research. Engberg et al. [17] reported that the addition of WW resulted in decreased pH of gizzard contents. Similarly, a significant reduction in the pH of gizzard contents of birds fed diets containing 20% WW was reported by Gabriel et al. [39]. The pH measured in gizzard was significantly lower in both WW and OH fed chickens than in chickens given control feed [19]. The logical explanation for the pH reduction is the increased gizzard volume resulting from the better functioning and stimulation of gizzard activity from fiber inclusion and thus a longer retention time, which allowed for more hydrochloric acid secretion in the proventriculus [50].

Another important aspect related to the gizzard development is the potential positive role of a functional gizzard in the control of bacterial populations. WW feeding has been reported to reduce the intestinal number of lactose-negative enterobacteria (i.e. *Salmonella* spp) as well as the number of *C*. *perfringens* [17]. These results indicate that a functional gizzard may act as a barrier organ preventing potential pathogenic bacteria from entering the distal GIT. Changes in pH of the GIT, especially in the upper part in addition to the barrier effect, may favor enzymatic activity, promoting nutrient digestibility and therefore may leave less nutrients available for bacterial proliferation in distal part of GIT [14]. Singh et al. [42] suggest that whole grains may encourage colonisation of commensal bacteria and discourage pathogenic and harmful bacteria in the intestinal tract through competitive exclusion, hydrochloric acid secretion, grinding action of gizzard or a combination of all these. Moen et al. [18] have shown that a stimulated gizzard delays the horizontal spread of *C*. *jejuni* in broiler flocks. In our experiment, both mash feeds and WW inclusion resulted in stimulated gizzard and reduced pH. As hypothesized, mash diets tended to reduce *C*. *jejuni* colonization when compared with pelleted diets, and WW inclusion at 7.5/15% reduced *C*. *jejuni* colonization when compared to lower and higher inclusion, but only 7 days post-infection, which confirms the delay in the spread of *C*. *jejuni*, at least with the lower rate of inclusion. In addition, the supplementation of 5% OH together with WW inclusion at 7.5/15% reduced cecal *C*. *jejuni* colonization in relation to the Control diet at slaughter age. However, the reductions obtained in our research were of limited magnitude.

To summarize, feed form had a large effect in broiler performance and GIT weight. Birds fed pelleted diets consumed more feed and gained more weight and exhibited better feed conversion that those fed mash diets. On the other hand, mash diets resulted in stronger and healthier gut organs, with increased development and lower pH. The WW inclusion had similar beneficial effects than mash diets, independently of the level of inclusion. However, there was no clear relationship between the described changes in the GIT and the level of *C*. *jejuni* counts at ceca level. Even so, some reductions in *C*. *jejuni* colonization were obtained with mash diets, the lower level of WW, and the combination of WW and OH.

In conclusion, despite of the clear changes observed in the GIT derived of the feed form and WW inclusion, no clear reductions in *C*. *jejuni* populations in the ceca were observed. However, WW and OH inclusion to mash diets significantly reduced cecal *C*. *jejuni* colonization at 42 days.
